# Supplementary material for: Quantifying ERK activity in response to inhibition of the BRAFV600E-MEK-ERK cascade using mathematical modelling
Source: Br J Cancer. 2021 Oct 7;125(11):1552–60. doi: 10.1038/s41416-021-01565-w (PMC8608797; doi:10.1038/s41416-021-01565-w)
Supplement: Supplementary file 1 — Supplementary Material [file 41416_2021_1565_MOESM1_ESM.pdf]

Supplementary Material – Quantifying ERK-activity in response to inhibition of the BRAFV600E-MEK-ERK cascade using mathematical modelling.

Sara Hamis,[1] Yury Kapelyukh,[2] Aileen McLaren,[2] Colin J. Henderson,[2] C. Roland Wolf,[2] Mark A.J. Chaplain.[1]

1 School of Mathematics and Statistics, University of St Andrews, St Andrews, Scotland, UK.
2 School of Medicine, Jacqui Wood Cancer Centre, Ninewells Hospital and Medical School, University of Dundee, Dundee, Scotland, UK.

Contents

SM1 Formulating the system of differential algebraic equations 2
SM1.1 The system of reactions (R.1-R.36) 2
SM1.2 The system of ordinary differential equations (O.1-O.36) 3
SM1.3 The conservation laws (C.1-C.7) 6

SM2 Code access and instructions 7
SM2.1 Code access - GitHub repository 7
SM2.2 Instructions on how to run the code 7
SM2.3 Instructions on how to modify the code 7
SM2.3.1 Modifying the rate constants 8
SM2.3.2 Modifying the chemical reactions/cascade structure 8
SM2.3.3 Modifying the initial conditions 8

SM3 Model parameters 8
SM3.1 Kinetic constants 8
SM3.2 Model initial condition (initial cascade component concentrations) 10

SM4 Data and derivation of model parameters 10
SM4.1 Rate constants for BRAF-ATP and BRAF-MEK reactions 10
SM4.2 Rate constants for MEK-ATP and MEK-ERK reactions 11
SM4.3 Rate constants for phosphatase reactions 12
SM4.4 Rate constants for drug reactions 12

SM5 Results not included in main manuscript 12
SM5.1 Extended time range dabrafenib responses 12

References 13

SM1 Formulating the system of differential algebraic equations

SM1.1 The system of reactions (R.1-R.36)

The system of chemical reactions (R.1-R.36), as listed below, describes signalling dynamics in the intracellular BRAFV600E-MEK-ERK cascade, when a cell is subjected to the BRAF-inhibitor dabrafenib and the MEK-inhibitor trametinib. The cascade and drug actions are schematically illustrated in Figures 1 and 2 in the main manuscript, respectively, and (R.1-R.36) are listed in Figure 3. The dot-notation $(\cdot)$ is here used to denote complexes of two or more molecles. Reactions involving dabrafenib and trametinib are respectively marked by single (*) and double (**) stars, and can be omitted if one wishes to study the system in the absence of these drugs.

System of reactions (R.1-R.36):

(R.1) $\mathrm{BRAF} + \mathrm{ATP} \underset{d_1}{\overset{a_1}{\rightleftharpoons}} \mathrm{BRAF{\cdot}ATP}$

(R.2) $\mathrm{BRAF} + \mathrm{MEK} \underset{d_2}{\overset{a_2}{\rightleftharpoons}} \mathrm{BRAF{\cdot}MEK}$

(R.3) $\mathrm{BRAF} + \mathrm{pMEK} \underset{d_2}{\overset{a_2}{\rightleftharpoons}} \mathrm{BRAF{\cdot}pMEK}$

(R.4) $\mathrm{BRAF{\cdot}ATP} + \mathrm{MEK} \underset{d_2}{\overset{a_2}{\rightleftharpoons}} \mathrm{BRAF{\cdot}ATP{\cdot}MEK} \xrightarrow{k_{12}} \mathrm{BRAF} + \mathrm{ADP} + \mathrm{pMEK}$

(R.5) $\mathrm{BRAF{\cdot}ATP} + \mathrm{pMEK} \underset{d_2}{\overset{a_2}{\rightleftharpoons}} \mathrm{BRAF{\cdot}ATP{\cdot}pMEK} \xrightarrow{k_{12}} \mathrm{BRAF} + \mathrm{ADP} + \mathrm{ppMEK}$

(R.6) $\mathrm{BRAF{\cdot}MEK} + \mathrm{ATP} \underset{d_1}{\overset{a_1}{\rightleftharpoons}} \mathrm{BRAF{\cdot}ATP{\cdot}MEK} \xrightarrow{k_{12}} \mathrm{BRAF} + \mathrm{ADP} + \mathrm{pMEK}$

(R.7) $\mathrm{BRAF{\cdot}pMEK} + \mathrm{ATP} \underset{d_1}{\overset{a_1}{\rightleftharpoons}} \mathrm{BRAF{\cdot}ATP{\cdot}pMEK} \xrightarrow{k_{12}} \mathrm{BRAF} + \mathrm{ADP} + \mathrm{ppMEK}$

(R.8) $\mathrm{pMEK} + \mathrm{phosph}_1 \underset{d_3}{\overset{a_3}{\rightleftharpoons}} \mathrm{pMEK{\cdot}phosph}_1 \xrightarrow{k_3} \mathrm{MEK} + \mathrm{phosph}_1$

(R.9) $\mathrm{ppMEK} + \mathrm{phosph}_1 \underset{d_3}{\overset{a_3}{\rightleftharpoons}} \mathrm{ppMEK{\cdot}phosph}_1 \xrightarrow{k_3} \mathrm{pMEK} + \mathrm{phosph}_1$

(R.10)* $\mathrm{BRAF} + \mathrm{DBF} \underset{d_4}{\overset{a_4}{\rightleftharpoons}} \mathrm{BRAF{\cdot}DBF}$

(R.11)* $\mathrm{BRAF{\cdot}MEK} + \mathrm{DBF} \underset{d_4}{\overset{a_4}{\rightleftharpoons}} \mathrm{BRAF{\cdot}MEK{\cdot}DBF}$

(R.12)* $\mathrm{BRAF{\cdot}pMEK} + \mathrm{DBF} \underset{d_4}{\overset{a_4}{\rightleftharpoons}} \mathrm{BRAF{\cdot}pMEK{\cdot}DBF}$

(R.13)* $\mathrm{BRAF{\cdot}DBF} + \mathrm{MEK} \underset{d_2}{\overset{a_2}{\rightleftharpoons}} \mathrm{BRAF{\cdot}MEK{\cdot}DBF}$

(R.14)* $\mathrm{BRAF{\cdot}DBF} + \mathrm{pMEK} \underset{d_2}{\overset{a_2}{\rightleftharpoons}} \mathrm{BRAF{\cdot}pMEK{\cdot}DBF}$

(R.15) $\mathrm{ppMEK} + \mathrm{ATP} \underset{d_5}{\overset{a_5}{\rightleftharpoons}} \mathrm{ppMEK{\cdot}ATP}$

(R.16) $\mathrm{ppMEK} + \mathrm{ERK} \underset{d_6}{\overset{a_6}{\rightleftharpoons}} \mathrm{ppMEK{\cdot}ERK}$

(R.17) $\mathrm{ppMEK} + \mathrm{pERK} \underset{d_6}{\overset{a_6}{\rightleftharpoons}} \mathrm{ppMEK{\cdot}pERK}$

(R.18) ppMEK·ATP + ERK $\underset{d_6}{\overset{a_6}{\rightleftharpoons}}$ ppMEK·ATP·ERK $\xrightarrow{k_{56}}$ ppMEK + ADP + pERK

(R.19) ppMEK·ATP + pERK $\underset{d_6}{\overset{a_6}{\rightleftharpoons}}$ ppMEK·ATP·pERK $\xrightarrow{k_{56}}$ ppMEK + ADP + ppERK

(R.20) ppMEK·ERK + ATP $\underset{d_5}{\overset{a_5}{\rightleftharpoons}}$ ppMEK·ATP·ERK $\xrightarrow{k_{56}}$ ppMEK + ADP + ppERK

(R.21) ppMEK·pERK + ATP $\underset{d_5}{\overset{a_5}{\rightleftharpoons}}$ ppMEK·ATP·pERK $\xrightarrow{k_{56}}$ ppMEK + ADP + ppERK

(R.22) pERK + $phosph_2$ $\underset{d_7}{\overset{a_7}{\rightleftharpoons}}$ pERK·$phosph_2$ $\xrightarrow{k_7}$ ERK + $phosph_2$

(R.23) ppERK + $phosph_2$ $\underset{d_7}{\overset{a_7}{\rightleftharpoons}}$ ppERK·$phosph_2$ $\xrightarrow{k_7}$ pERK + $phosph_2$

(R.24)** ppMEK + TMT $\underset{d_8}{\overset{a_8}{\rightleftharpoons}}$ ppMEK·TMT

(R.25)** ppMEK·ATP + TMT $\underset{d_8}{\overset{a_8}{\rightleftharpoons}}$ ppMEK·ATP·TMT

(R.26)** ppMEK·ERK + TMT $\underset{d_8}{\overset{a_8}{\rightleftharpoons}}$ ppMEK·ERK·TMT

(R.27)** ppMEK·pERK + TMT $\underset{d_8}{\overset{a_8}{\rightleftharpoons}}$ ppMEK·pERK·TMT

(R.28)** ppMEK·ERK·ATP + TMT $\underset{d_8}{\overset{a_8}{\rightleftharpoons}}$ ppMEK·ERK·ATP·TMT

(R.29)** ppMEK·pERK·ATP + TMT $\underset{d_8}{\overset{a_8}{\rightleftharpoons}}$ ppMEK·pERK·ATP·TMT

(R.30)** ppMEK·TMT + ATP $\underset{d_5}{\overset{a_5}{\rightleftharpoons}}$ ppMEK·ATP·TMT

(R.31)** ppMEK·TMT + ERK $\underset{d_6}{\overset{a_6}{\rightleftharpoons}}$ ppMEK·ERK·TMT

(R.32)** ppMEK·TMT + pERK $\underset{d_6}{\overset{a_6}{\rightleftharpoons}}$ ppMEK·pERK·TMT

(R.33)** ppMEK·TMT·ERK + ATP $\underset{d_5}{\overset{a_5}{\rightleftharpoons}}$ ppMEK·ERK·ATP·TMT

(R.34)** ppMEK·TMT·pERK + ATP $\underset{d_5}{\overset{a_5}{\rightleftharpoons}}$ ppMEK·pERK·ATP·TMT

(R.35)** ppMEK·TMT·ATP + ERK $\underset{d_6}{\overset{a_6}{\rightleftharpoons}}$ ppMEK·ERK·ATP·TMT

(R.36)** ppMEK·TMT·ATP + pERK $\underset{d_6}{\overset{a_6}{\rightleftharpoons}}$ ppMEK·pERK·ATP·TMT

SM1.2 The system of ordinary differential equations (O.1-O.36)

Using the law of mass action, the system of reactions (R.1-R.36) can be formulated in terms of a system of ordinary differential equations (O.1-O.36). We here let $\mathbf{y_{36x1}}$ denote a column vector with 36 elements, where each element corresponds to a signalling molecule concentration so that,

$$
\begin{aligned}
&y(1) = [BRAF],\\
&y(2) = [ATP],\\
&y(3) = [ATP \cdot BRAF],\\
&y(4) = [MEK],
\end{aligned}
$$

$$
\begin{aligned}
y(5) &= [BRAF \cdot MEK],\\
y(6) &= [pMEK],\\
y(7) &= [BRAF \cdot pMEK],\\
y(8) &= [ATP \cdot BRAF \cdot MEK],\\
y(9) &= [ADP],\\
y(10) &= [ATP \cdot BRAF \cdot pMEK],\\
y(11) &= [ppMEK],\\
y(12) &= [phosph_1],\\
y(13) &= [pMEK \cdot phosph_1],\\
y(14) &= [phosph_1 \cdot ppMEK],\\
y(15) &= [DBF],\\
y(16) &= [BRAF \cdot DBF],\\
y(17) &= [BRAF \cdot DBF \cdot MEK],\\
y(18) &= [BRAF \cdot DBF \cdot pMEK],\\
y(19) &= [ATP \cdot ppMEK],\\
y(20) &= [ERK],\\
y(21) &= [ERK \cdot ppMEK],\\
y(22) &= [pERK],\\
y(23) &= [pERK \cdot ppMEK],\\
y(24) &= [ATP \cdot ERK \cdot ppMEK],\\
y(25) &= [ATP \cdot pERK \cdot ppMEK],\\
y(26) &= [ppERK],\\
y(27) &= [phosph_2],\\
y(28) &= [pERK \cdot phosph_2],\\
y(29) &= [phosph_2 \cdot ppERK],\\
y(30) &= [TMT],\\
y(31) &= [TMT \cdot ppMEK],\\
y(32) &= [ATP \cdot TMT \cdot ppMEK],\\
y(33) &= [ERK \cdot TMT \cdot ppMEK],\\
y(34) &= [TMT \cdot pERK \cdot ppMEK],\\
y(35) &= [ATP \cdot ERK \cdot TMT \cdot ppMEK],\\
y(36) &= [ATP \cdot TMT \cdot pERK \cdot ppMEK].
\end{aligned}
$$

Using this notation, the system of ODEs (O.1-O.36) can be formulated as,

$$
\begin{aligned}
\frac{dy(1)}{dt} = {} & y(1)y(2)(-a_1) + d_1y(3) + y(1)y(4)(-a_2) + d_2y(5) + y(1)y(6)(-a_2) + d_2y(7)\\
& + y(8)k_{12} + y(10)k_{12} + y(8)k_{12} + y(10)k_{12} + y(1)y(15)(-a_4) + d_4y(16),
\end{aligned} \tag{O.1}
$$

$$
\begin{aligned}
\frac{dy(2)}{dt} = {} & y(1)y(2)(-a_1) + d_1y(3) + y(5)y(2)(-a_1) + d_1y(8) + y(7)y(2)(-a_1) + d_1y(10)\\
& + y(11)y(2)(-a_5) + d_5y(19) + y(21)y(2)(-a_5) + d_5y(24) + y(23)y(2)(-a_5)\\
& + d_5y(25) + y(31)y(2)(-a_5) + d_5y(32) + y(33)y(2)(-a_5) + d_5y(35)\\
& + y(34)y(2)(-a_5) + d_5y(36),
\end{aligned} \tag{O.2}
$$

$$
\frac{dy(3)}{dt} = y(3)(-d_1) + a_1(y(1)y(2)) + y(3)y(4)(-a_2) + d_2y(8) + y(3)y(6)(-a_2) + d_2y(10), \tag{O.3}
$$

$$
\begin{aligned}
\frac{dy(4)}{dt} = {} & y(1)y(4)(-a_2) + d_2y(5) + y(3)y(4)(-a_2) + d_2y(8) + y(13)k_3 + y(16)y(4)(-a_2)\\
& + d_2y(17),
\end{aligned} \tag{O.4}
$$

$$
\frac{dy(5)}{dt} = y(5)(-d_2) + a_2(y(1)y(4)) + y(5)y(2)(-a_1) + d_1y(8) + y(5)y(15)(-a_4) + d_4y(17), \tag{O.5}
$$

$$\frac{dy(6)}{dt} = y(1)y(6)(-a_2) + d_2y(7) + y(8)k_{12} + y(3)y(6)(-a_2) + d_2y(10) + y(8)k_{12} + y(6)y(12)(-a_3) + d_3y(13) + y(14)k_3 + y(16)y(6)(-a_2) + d_2y(18), \tag{O.6}$$

$$\frac{dy(7)}{dt} = y(7)(-d_2) + a_2(y(1)y(6)) + y(7)y(2)(-a_1) + d_1y(10) + y(7)y(15)(-a_4) + d_4y(18), \tag{O.7}$$

$$\frac{dy(8)}{dt} = y(8)(-d_2 - k_{12}) + a_2(y(3)y(4)) + y(8)(-d_1 - k_{12}) + a_1(y(5)y(2)), \tag{O.8}$$

$$\frac{dy(9)}{dt} = y(8)k_{12} + y(10)k_{12} + y(8)k_{12} + y(10)k_{12} + y(24)k_{56} + y(25)k_{56} + y(24)k_{56} + y(25)k_{56}, \tag{O.9}$$

$$\frac{dy(10)}{dt} = y(10)(-d_2 - k_{12}) + a_2(y(3)y(6)) + y(10)(-d_1 - k_{12}) + a_1(y(7)y(2)), \tag{O.10}$$

$$\frac{dy(11)}{dt} = y(10)k_{12} + y(10)k_{12} + y(11)y(12)(-a_3) + d_3y(14) + y(11)y(2)(-a_5) + d_5y(19) + y(11)y(20)(-a_6) + d_6y(21) + y(11)y(22)(-a_6) + d_6y(23) + y(24)k_{56} + y(25)k_{56} + y(24)k_{56} + y(25)k_{56} + y(11)y(30)(-a_8) + d_8y(31), \tag{O.11}$$

$$\frac{dy(12)}{dt} = y(6)y(12)(-a_3) + d_3y(13) + y(13)k_3 + y(11)y(12)(-a_3) + d_3y(14) + y(14)k_3, \tag{O.12}$$

$$\frac{dy(13)}{dt} = y(13)(-d_3 - k_3) + a_3(y(6)y(12)), \tag{O.13}$$

$$\frac{dy(14)}{dt} = y(14)(-d_3 - k_3) + a_3(y(11)y(12)), \tag{O.14}$$

$$\frac{dy(15)}{dt} = y(1)y(15)(-a_4) + d_4y(16) + y(5)y(15)(-a_4) + d_4y(17) + y(7)y(15)(-a_4) + d_4y(18), \tag{O.15}$$

$$\frac{dy(16)}{dt} = y(16)(-d_4) + a_4(y(1)y(15)) + y(16)y(4)(-a_2) + d_2y(17) + y(16)y(6)(-a_2) + d_2y(18), \tag{O.16}$$

$$\frac{dy(17)}{dt} = y(17)(-d_4) + a_4(y(5)y(15)) + y(17)(-d_2) + a_2(y(16)y(4)), \tag{O.17}$$

$$\frac{dy(18)}{dt} = y(18)(-d_4) + a_4(y(7)y(15)) + y(18)(-d_2) + a_2(y(16)y(6)), \tag{O.18}$$

$$\frac{dy(19)}{dt} = y(19)(-d_5) + a_5(y(11)y(2)) + y(19)y(20)(-a_6) + d_6y(24) + y(19)y(22)(-a_6) + d_6y(25) + y(19)y(30)(-a_8) + d_8y(32), \tag{O.19}$$

$$\frac{dy(20)}{dt} = y(11)y(20)(-a_6) + d_6y(21) + y(19)y(20)(-a_6) + d_6y(24) + y(28)k_7 + y(31)y(20)(-a_6) + d_6y(33) + y(32)y(20)(-a_6) + d_6y(35), \tag{O.20}$$

$$\frac{dy(21)}{dt} = y(21)(-d_6) + a_6(y(11)y(20)) + y(21)y(2)(-a_5) + d_5y(24) + y(21)y(30)(-a_8) + d_8y(33), \tag{O.21}$$

$$\frac{dy(22)}{dt} = y(11)y(22)(-a_6) + d_6y(23) + y(24)k_{56} + y(19)y(22)(-a_6) + d_6y(25) + y(24)k_{56} + y(22)y(27)(-a_7) + d_7y(28) + y(29)k_7 + y(31)y(22)(-a_6) + d_6y(34) + y(32)y(22)(-a_6) + d_6y(36), \tag{O.22}$$

$$\frac{dy(23)}{dt} = y(23)(-d_6) + a_6(y(11)y(22)) + y(23)y(2)(-a_5) + d_5y(25) + y(23)y(30)(-a_8) + d_8y(34), \tag{O.23}$$

$$\frac{dy(24)}{dt} = y(24)(-d_6 - k_{56}) + a_6(y(19)y(20)) + y(24)(-d_5 - k_{56}) + a_5(y(21)y(2)) + y(24)y(30)(-a_8) + d_8y(35), \tag{O.24}$$

$$\frac{dy(25)}{dt} = y(25)(-d_6 - k_{56}) + a_6(y(19)y(22)) + y(25)(-d_5 - k_{56}) + a_5(y(23)y(2)) + y(25)y(30)(-a_8) + d_8y(36), \tag{O.25}$$

$$\frac{dy(26)}{dt} = y(25)k_{56} + y(25)k_{56} + y(26)y(27)(-a_7) + d_7y(29), \tag{O.26}$$

$$\frac{dy(27)}{dt} = y(22)y(27)(-a_7) + d_7y(28) + y(28)k_7 + y(26)y(27)(-a_7) + d_7y(29) + y(29)k_7, \tag{O.27}$$

$$\frac{dy(28)}{dt} = y(28)(-d_7 - k_7) + a_7(y(22)y(27)), \tag{O.28}$$

$$\frac{dy(29)}{dt} = y(29)(-d_7 - k_7) + a_7(y(26)y(27)), \tag{O.29}$$

$$\frac{dy(30)}{dt} = y(11)y(30)(-a_8) + d_8y(31) + y(19)y(30)(-a_8) + d_8y(32) + y(21)y(30)(-a_8) + d_8y(33) + y(23)y(30)(-a_8) + d_8y(34) + y(24)y(30)(-a_8) + d_8y(35) + y(25)y(30)(-a_8) + d_8y(36), \tag{O.30}$$

$$\frac{dy(31)}{dt} = y(31)(-d_8) + a_8(y(11)y(30)) + y(31)y(2)(-a_5) + d_5y(32) + y(31)y(20)(-a_6) + d_6y(33) + y(31)y(22)(-a_6) + d_6y(34), \tag{O.31}$$

$$\frac{dy(32)}{dt} = y(32)(-d_8) + a_8(y(19)y(30)) + y(32)(-d_5) + a_5(y(31)y(2)) + y(32)y(20)(-a_6) + d_6y(35) + y(32)y(22)(-a_6) + d_6y(36), \tag{O.32}$$

$$\frac{dy(33)}{dt} = y(33)(-d_8) + a_8(y(21)y(30)) + y(33)(-d_6) + a_6(y(31)y(20)) + y(33)y(2)(-a_5) + d_5y(35), \tag{O.33}$$

$$\frac{dy(34)}{dt} = y(34)(-d_8) + a_8(y(23)y(30)) + y(34)(-d_6) + a_6(y(31)y(22)) + y(34)y(2)(-a_5) + d_5y(36), \tag{O.34}$$

$$\frac{dy(35)}{dt} = y(35)(-d_8) + a_8(y(24)y(30)) + y(35)(-d_5) + a_5(y(33)y(2)) + y(35)(-d_6) + a_6(y(32)y(20)), \tag{O.35}$$

$$\frac{dy(36)}{dt} = y(36)(-d_8) + a_8(y(25)y(30)) + y(36)(-d_5) + a_5(y(34)y(2)) + y(36)(-d_6) + a_6(y(32)y(22)). \tag{O.36}$$

SM1.3 The conservation laws (C.1-C.7)

The regarded system described by (O.1-O.36) obeys a set of conservation laws (C.1-C.7) which are,

$$0 = y(1) + y(3) + y(5) + y(7) + y(8) + y(10) + y(16) + y(17) + y(18) - BRAF_{tot}, \tag{C.1}$$

$$0 = y(4) + y(5) + y(6) + y(7) + y(8) + y(10) + y(11) + y(13) + y(14) + y(17) + y(18) + y(19) + y(21) + y(23) + y(24) + y(25) + y(31) + y(32) + y(33) + y(34) + y(35) + y(36) - MEK_{tot}, \tag{C.2}$$

$$0 = y(20) + y(21) + y(22) + y(23) + y(24) + y(25) + y(26) + y(28) + y(29) + y(33) + y(34) + y(35) + y(36) - ERK_{tot}, \tag{C.3}$$

$$0 = y(2) + y(3) + y(8) + y(10) + y(19) + y(24) + y(25) + y(32) + y(35) + y(36) - ATP_{tot}, \quad (C.4)$$
$$0 = y(15) + y(16) + y(17) + y(18) - DBF_{tot}, \quad (C.5)$$
$$0 = y(30) + y(31) + y(32) + y(33) + y(34) + y(35) + y(36) - TMT_{tot}, \quad (C.6)$$
$$0 = y(12) + y(13) + y(14) - phosph_{1,tot}, \quad (C.7)$$
$$0 = y(27) + y(28) + y(29) - phosph_{2,tot}. \quad (C.8)$$

Note that, we assume a cellular ADP-to-ATP recycling to continually occur and thus the $[ADP]$ variable is not included in the $[ATP]$ conservation law. This ATP-to-ADP recycling is not explicitly included in the model. In C.1-C.7, the total concentrations, denoted by a tot-subscript, are equal to the initial concentrations described in Table ST2 (Section SM3.2) so that,

$$\begin{aligned} BRAF_{tot} &= \text{[BRAF](0)}, \\ MEK_{tot} &= \text{[MEK](0)}, \\ ERK_{tot} &= \text{[ERK](0)}, \\ phosph_{1,tot} &= [\text{phosph}_1](0), \\ phosph_{2,tot} &= [\text{phosph}_2](0), \\ DBF_{tot} &= \text{[DBF](0)}, \\ TMT_{tot} &= \text{[TMT](0)}. \end{aligned}$$

SM2 Code access and instructions

The computational MATLAB [1] code includes functions to convert the system of reactions (R.1-R.36) into a system of differential algebraic equations, *i.e.,* (O.1-O.36) with code-generated substitutions (C.1-C.7), using the law of mass action. The MATLAB function `ode15s` is then used to solve for $\mathbf{y_{36x1}}$ (*i.e.*, the signalling molecule concentration vector) as a function of system concentrations and time.

SM2.1 Code access - GitHub repository

The MATLAB code, developed for this project, is available on the code hosting platform GitHub, in the project repository (https://github.com/SJHamis/MAPKcascades).

SM2.2 Instructions on how to run the code

The model outputs/result plots presented in the main manuscript can be generated by running the five main-files, with file names `main_[insert option].m`. The code includes three sub-directories: (1) `auxiliary_files_plots`, which includes files needed to generate data for specific time points, (2) `auxiliary_files_model_setup`, which includes files needed to set up the DAE MATLAB function file (`mapk_cascade_DAE.m`), (3) `model_details`, in which the system of reactions (R.1-R.36) and the rate constants are manually set.

SM2.3 Instructions on how to modify the code

Instructions on how to edit the cascade structure, rate constants and initial conditions are provided below. For more detailed information, see in-line comments in the MATLAB code.

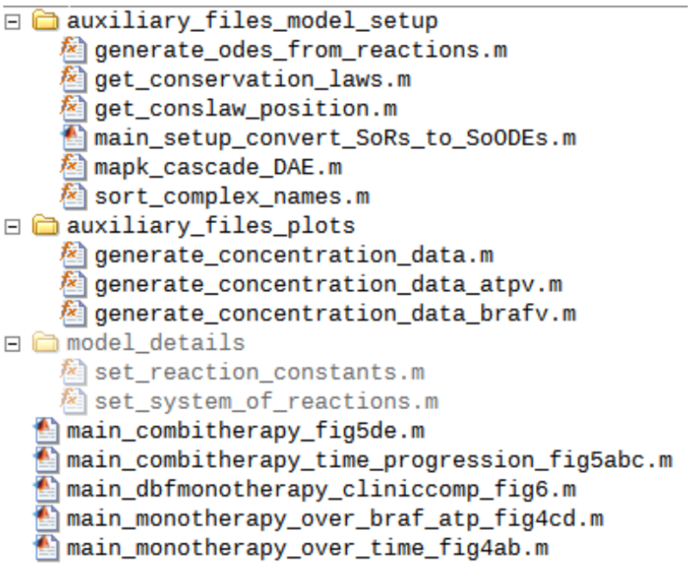

Figure SF1: Code directories.

SM2.3.1 Modifying the rate constants

The rate constants can be manually edited in the file
`model_details/set_reaction_constants.m`.
By first editing the constants and thereafter running the file
`auxiliary_files_model_setup/main_setup_convert_SoRs_to_SoODEs.m`,
the MATLAB DAE function file
`auxiliary_files_model_setup/mapk_cascade_DAE.m`
will be updated to include the modified rate constants.

SM2.3.2 Modifying the chemical reactions/cascade structure

The system of reactions can be manually edited in the file
`model_details/set_system_of_reactions.m`.
By first editing the system of reactions, by for example by including more terms, and thereafter running the file
`auxiliary_files_model_setup/main_setup_convert_SoRs_to_SoODEs.m`,
the function files
`auxilary_files_model_setup/mapk_cascade_DAE.m`
and
`auxilary_files_model_setup/get_conslaw_position.m`
will be updated to include the new model equations (ODEs and conservation laws).

SM2.3.3 Modifying the initial conditions

The model initial condition (*i.e.,* the initial cascade component concentrations) can be modified in the main files,
`main_[insert option].m`.

SM3 Model parameters

SM3.1 Kinetic constants

Rate constants in the system of reactions (R.1-R.36)				
constant	value	reference	description	appearance
a_1	0.106μM^{-1}s^{-1}	[2, 3]	Forward rate constant for ATP binding to BRAF (or a BRAF complex with MEK/pMEK).	R.1,R.6,R.7
d_1	6.23 s^{-1}	[2, 3, 4]	Reverse rate constant for ATP binding to BRAF (or a BRAF complex with MEK/pMEK).	R.1,R.6,R.7
a_2	0.106μM^{-1}s^{-1}	†	Forward rate constant for MEK/pMEK binding to BRAF (or a BRAF complex with ATP/DBF).	R.2,R.3,R.4, R.5,R.13,R.14
d_2	0.02385 s^{-1}	[2, 3]	Reverse rate constant for MEK/pMEK binding to BRAF (or a BRAF complex with ATP/DBF).	R.2,R.3,R.4, R.5,R.13,R.14
$k_{1,2}$	0.66 s^{-1}	[3]	Catalytic rate constant for production of pMEK/ppMEK from BRAF·ATP complex with MEK/pMEK.	R.4,R.5,R.6, R.7
a_3	0.106μM^{-1}s^{-1}	†	Forward rate constant for phosph$_1$ binding to pMEK/ppMEK.	R.8,R.9
d_3	0.0159 s^{-1}	[2, 3]	Reverse rate constant for phosph$_1$ binding to pMEK/ppMEK.	R.8,R.9
k_3	0.0159 s^{-1}	[2, 3]	Catalytic rate constant for production of MEK/pMEK from phosph$_1$ complex with pMEK/ppMEK.	R.8,R.9
a_4	0.106μM^{-1}s^{-1}	†	Forward rate constant for the drug DBF binding to BRAF (or a BRAF complex with MEK/pMEK).	R.10,R.11,R.12
d_4	0.0000593 s^{-1}	[2, 3, 5, 6, 7, 8]	Reverse rate constant for the drug DBF binding to BRAF (or a BRAF complex with MEK/pMEK).	R.10,R.11,R.12
a_5	0.106μM^{-1}s^{-1}	†	Forward rate constant for ATP binding to ppMEK (or a ppMEK complex with ERK/pERK/TMT/TMT&ERK/TMT&pERK).	R.15,R.20,R.21, R.30,R.33,R.34
d_5	0.3468 s^{-1}	[2, 3, 9]	Reverse rate constant for ATP binding to ppMEK (or a ppMEK complex with ERK/pERK/TMT/TMT&ERK/TMT&pERK).	R.15,R.20,R.21, R.30,R.33,R.34
a_6	0.106μM^{-1}s^{-1}	†	Forward rate constant for ERK/pERK binding to ppMEK (or a ppMEK complex with ATP/TMT/TMT&ATP).	R.16,R.17,R.18, R.19,R.31,R.32, R.35,R.36
d_6	0.01184 s^{-1}	[2, 3, 9]	Reverse rate constant for ERK/pERK binding to ppMEK (or a ppMEK complex with ATP/TMT/TMT&ATP).	R.16,R.17,R.18, R.19,R.31,R.32, R.35,R.36
$k_{5,6}$	0.0242 s^{-1}	[9]	Catalytic rate constant for production of pERK/ppERK from ppMEK·ATP complex with ERK/pERK.	R.18,R.19,R.20, R.21
a_7	0.106μM^{-1}s^{-1}	†	Forward rate constant for phosph$_2$ binding to pERK/ppERK.	R.22,R.23
d_7	0.0159 s^{-1}	[2, 3]	Reverse rate constant for phosph$_2$ binding to pERK/ppERK.	R.22,R.23
k_7	0.0159 s^{-1}	[2, 3]	Catalytic rate constant for production of ERK/pERK from phosph$_2$ complex with pERK/ppERK.	R.22,R.23
a_8	0.106μM^{-1}s^{-1}	†	Forward rate constant for the drug TMT binding to ppMEK (or a ppMEK complex with ATP/ERK/pERK/ERK&ATP/pERK&ATP).	R.24,R.25,R.26, R.27,R.28,R.29
d_8	0.0012296 s^{-1}	[2, 3, 10]	Reverse rate constant for the drug TMT binding to ppMEK (or a ppMEK complex with ATP/ERK/pERK/ERK&ATP/pERK&ATP).	R.24,R.25,R.26, R.27,R.28,R.29

Table ST1: A table of the rate constants appearing in the system of reactions (R.1-R.36). The system includes eight forward rate constants $a_1, a_2, .., a_8$, eight reverse rate constants $d_1, d_2, .., d_8$, and four catalytic rate constants $k_{1,2}, k_3, k_{5,6}, k_7$. Stroke symbols (/) in the description column denote 'or', whilst ampersand symbols (&) denote 'and'. The rightmost column describes in which of the reactions (R.1-R.36) the constants appear. † All forward rate constants are of the same value.

SM3.2 Model initial condition (initial cascade component concentrations)

Initial concentrations		
Initial condition	value	reference
[BRAF](0)	3-10 nM*	[2]
[MEK](0)	1.2 μM	[2]
[ERK](0)	1.2 μM	[2]
[phosph1](0)	0.3 nM	[2]
[phosph2](0)	0.12 μM	[2]
[ATP](0)	1-5 mM**	[9]
[DBF](0)	*applied DBF dose* (here 0-10 μM)	
[TMT](0)	*applied TMT dose* (here 0-10 μM)	

Table ST2: Initial model concentrations, where the (0) notation denotes 'at time zero'. For molecule concentrations not listed in this table, the initial conditions is 0 μM. *Huang & Ferrell [2] listed MAPKKK=3nM, and we use this as our baseline value. However, in order to study amplified BRAF levels as a mode of drug resistance, we explore initial BRAF concentrations within the range 3-10 nM. Similarly, initial ATP concentrations are explored within the range 1-5mM, but the baseline value is 1mM.

SM4 Data and derivation of model parameters

All model parameter values for the forward rate constants $a_1, a_2, ..., a_8$, the reverse rate constants $d_1, d_2, ..., d_8$ and the catalytic rate constant $k_{1,2}, k_3, k_{5,6}, k_7$ are obtained from data that is available in the literature. Note that $a_i = a_1 \forall i = 2, 3, ...8$, as is explained in the main manuscript (in **Methods - Model parameters**).

SM4.1 Rate constants for BRAF-ATP and BRAF-MEK reactions

VanScyoc *et al.* [3] provide kinetic data for the phosphorylation of the substrate MEK by wild type BRAF. We use their data to approximate the parameter values for a_1, d_2 and $k_{1,2}$ in our model, *i.e.* rate constants for the BRAF-ATP and BRAF-MEK reactions. We also compute the reverse BRAF-ATP rate constant for wild type BRAF, here denoted by $d_{1,WT}$. We then use $d_{1,WT}$ to find a_1, as the catalytic rate constant $k_{1,2}$ and the forward rate constant a_1, can be assumed to be the same for both V600E-mutant and wild type BRAF interacting with ATP. In order to set d_1, we thereafter use data for $BRAF^{V600E}$-ATP interactions provided by Hatzivassiliou *et al.* [4].

BRAF-ATP reactions (approximating $a_1, d_1, k_{1,2}$): VanScyoc *et al.* report that the disassociation constant for ATP binding to wild type BRAF is $K_{d1,WT}$(ATP) $= 7.8 \pm 2.2\mu$M. We use this K_d-value to find the relationship between a_1 and $d_{1,WT}$ using,

$$K_{d1,WT} = \frac{d_{1,WT}}{a_1} \approx 7.8\mu\text{M} \Longrightarrow d_{1,WT} \approx 7.8\mu\text{M} \cdot a_1. \quad \text{(P.1)}$$

VanScyoc *et al.* further report the catalytic rate constant for BRAF-ATP reactions to be $k_{cat} = 0.66 \pm 0.30\text{s}^{-1}$, which we can directly use as a parameter value for $k_{1,2}$ so that

$$k_{1,2} \approx 0.66\text{s}^{-1}. \quad \text{(P.2)}$$

We approximate the Michaelis constant, $K_{m1,WT}$, as the average value of K_m(ATP) at 0.15 and 0.6 μM MEK1, reported by VanScyoc *et al.*, so that $K_{m1,WT} \approx 14\mu$M. We can now use (P.1), (P.2) and

the data for the Michaelis constant to solve for the parameter value of a_1,

$$K_{m1,WT} = \frac{d_{1,WT} + k_{1,2}}{a_1} \Longrightarrow a_1 \cdot K_{m1,WT} = d_{1,WT} + k_{1,2} \Longrightarrow a_1 \cdot 14\mu\text{M} \approx (7.8\mu\text{M} \cdot a_1) + 0.66\text{s}^{-1} \Longrightarrow$$

$$\Longrightarrow a_1(14\mu\text{M} - 7.8\mu\text{M}) \approx 0.66\text{s}^{-1} \Longrightarrow a_1 \cdot 6.2\mu\text{M} \approx 0.66\text{s}^{-1} \Longrightarrow a_1 \approx \frac{0.66\text{s}^{-1}}{6.2\mu\text{M}} = 0.106\text{s}^{-1}(\mu\text{M})^{-1}. \tag{P.3}$$

If we now plug this value of a_1 into the expression for $d_{1,WT}$ in (P.1) we obtain,

$$d_{1,WT} \approx 7.8\mu\text{M} \cdot a_1 = 7.8\mu\text{M} \cdot 0.106\text{s}^{-1}(\mu\text{M})^{-1} = 0.8268\text{s}^{-1}. \tag{P.4}$$

In order to now find d_1, the reverse rate constant for V600E mutant BRAF, we use the ATP-BRAF$^{\text{V600E}}$ K_m value reported by Hatzivassiliou *et al.* which is 65 μM. Using this, we can obtain d_1 via

$$K_{m,1} = \frac{d_1 + k_{1,2}}{a_1} \Longrightarrow d_1 = K_{m,1}a_1 - k_{1,2} = 65\mu\text{M} \cdot 0.106\text{s}^{-1}(\mu\text{M})^{-1} - 0.66\text{s}^{-1} = 6.23\text{s}^{-1}. \tag{P.5}$$

Hatzivassiliou *et al.* further report that the ATP-BRAF K_m value is 5μM for wild type BRAF, which is in good agreement with VanScyoc *et al.*'s data.

BRAF-MEK reactions (approximating d_2): From VanScyoc *et al.*'s data, we can approximate the disassociation constant $K_{d2} = d_2/a_2$ as $K_{0.5}$(MEK1) $\approx 0.225\mu$M, where this value is the average of the reported $K_{0.5}$ values at 10 and 100 μM ATP. As $a_2 = a_1$, we can now find d_2 via

$$K_{0.5} = K_{d2} = \frac{d_2}{a_2} \approx 0.225\mu\text{M} \Longrightarrow d_2 \approx 0.225\mu\text{M} \cdot a_2 = 0.225\mu\text{M} \cdot a_1 \approx \tag{P.6}$$
$$\approx 0.225\mu\text{M} \cdot 0.106s^{-1}(\mu\text{M})^{-1} = 0.02385s^{-1}.$$

Note that the catalytic rate constant for BRAF-MEK, and ATP, reactions was obtained in (P.2).

SM4.2 Rate constants for MEK-ATP and MEK-ERK reactions

Mansour *et al.* [9] provide steady-state rate constants for MEK reactions. We use data from their study to set model parameter values describing MEK-ATP and MEK-ERK reactions.

MEK-ATP interactions (approximating $d_5, k_{5,6}$): Mansour *et al.* report that $V_m = 1.45 \pm 0.05\text{min}^{-1}$. After re-expressing this value in units of 'per second', we can directly use this data to approximate our parameter value of $k_{5,6}$,

$$k_{5,6} \approx 0.0242\text{s}^{-1}. \tag{P.7}$$

Mansour *et al.* further report that $K_{m,ATP} = 3.5 \pm 0.8\mu$M, which corresponds to K_{m5} in our model so that,

$$K_{m5} = \frac{d_5 + k_{5,6}}{a_5} \approx 3.5\mu\text{M} \Longrightarrow \frac{d_5 + 0.0242\text{s}^{-1}}{a_5} \approx 3.5\mu\text{M} \Longrightarrow d_5 \approx 3.5\mu\text{M} \cdot a_5 - 0.0242\text{s}^{-1}, \tag{P.8}$$

where we have used (P.7). By further using the convention that $a_5 = a_1 \approx 0.106s^{-1}(\mu\text{M})^{-1}$, we obtain a value for d_5 such that,

$$d_5 \approx 3.5\mu\text{M} \cdot a_1 - 0.0242\text{s}^{-1} \approx 3.5\mu\text{M} \cdot 0.106\text{s}^{-1}(\mu\text{M})^{-1} - 0.0242\text{s}^{-1} = 0.3468\text{s}^{-1}. \tag{P.9}$$

MEK-ERK interactions (approximating d_6): From Mansour *et al.*, the Michaelis constant for K_{m6} can be found, as $K_{m,ERK2} = 0.34 \pm 0.06\mu$M and thus

$$K_{m6} = \frac{d_6 + k_{5,6}}{a_6} \approx 0.34\mu\text{M}. \tag{P.10}$$

As $a_6 = a_1$ and $k_{5,6}$ have already been set, we can now solve for d_6,

$$\frac{d_6 + k_{5,6}}{a_1} \approx 0.34\mu\text{M} \Longrightarrow \frac{d_6 + 0.0242\text{s}^{-1}}{0.106 s^{-1}(\mu\text{M})^{-1}} \approx 0.34\mu\text{M} \Longrightarrow d_6 \approx 0.34\mu\text{M} \cdot 0.106 s^{-1}(\mu\text{M})^{-1} - 0.0242\text{s}^{-1} = \tag{P.11}$$
$$= 0.01184\text{s}^{-1}.$$

SM4.3 Rate constants for phosphatase reactions

phosph₁ and phosph₁ reactions (approximating d_3, k_3, d_7, k_7): Huang & Ferrell [2] estimated that, for all reactions,

$$K_m = \frac{d+k}{a} = 0.3\mu\text{M}. \tag{P.12}$$

We use this expression for the phosphatase reactions $i = 3$ and $i = 7$, so that

$$d_{3/7} + k_{3/7} = 0.3\mu\text{M} \cdot a_{3/7}, \tag{P.13}$$

where the slash notation (/) denotes *'or'*. By further estimating that $d_3 = k_3$ and $d_7 = k_7$, we obtain

$$d_{3/7} = k_{3/7} = \frac{0.3\mu\text{M} \cdot a_{3/7}}{2} = 0.15\mu\text{M} \cdot a_{3/7}, \tag{P.14}$$

and with $a_{3/7} = a_1$ we get,

$$d_{3/7} = k_{3/7} = 0.15\mu\text{M} \cdot a_1 \approx 0.15\mu\text{M} \cdot 0.106\text{s}^{-1}(\mu\text{M})^{-1} \approx 0.0159\text{s}^{-1}. \tag{P.15}$$

SM4.4 Rate constants for drug reactions

BRAF-DBF interactions (approximating d_4): Data pertaining to DBF reactions is gathered from the US Food and Drug Administration [5], according to which the *in vitro* IC50 value is 0.65nM. Rheault *et al.* [6] similarly report this IC50 value to be 0.7nM. We use this IC50 value to estimate the inhibitory constant K_i via the Cheng-Prusoff approach so that,

$$K_i = \frac{\text{IC50}}{1 + [L]/K_d} = \frac{0.65\text{nM}}{1 + 10\mu\text{M}/65\mu\text{M}} = 0.56\text{nM}, \tag{P.16}$$

where the experimental ligand (ATP) concentration is 10μM [7], and the ATP K_d value has been approximated as $K_m = 65\mu$M, as previously used. We can now find d_4 via,

$$K_{i4} = \frac{d_4}{a_4} = 0.00056\mu\text{M} \Longrightarrow d_4 = 0.00056\mu\text{M} \cdot a_4. \tag{P.17}$$

With $a_4 = a_1 \approx 0.106\text{s}^{-1}(\mu\text{M})^{-1}$, the value of d_4 can now be obtained as

$$d_4 = 0.00056\mu\text{M} \cdot a_1 \approx 0.00056\mu\text{M} \cdot 0.106\text{s}^{-1}(\mu\text{M})^{-1} = 5.93 \cdot 10^{-5} s^{-1}. \tag{P.18}$$

Koelblinger *et al.* [8] report the reverse rate constant for dabrafenib-BRAF interactions to be $9.6 \cdot 10^{-5} s^{-1}$, which is in good agreement with our calculated value.

MEK-TMT interactions (approximating d_8): TMT data is gathered from Gilmartin *et al.* [10] who report that $K_i = 0.0116 \pm 0.0007\mu$M. With the median as our K_{d8} value, we obtain

$$K_{d8} = \frac{d_8}{a_8} \Longrightarrow d_8 = 0.0116\mu\text{M} \cdot a_8 = 0.0116\mu\text{M} \cdot a_1 \approx 0.0116\mu\text{M} \cdot 0.106\text{s}^{-1}(\mu\text{M})^{-1} = 0.0012296\text{s}^{-1}. \tag{P.19}$$

SM5 Results not included in main manuscript

SM5.1 Extended time range dabrafenib responses

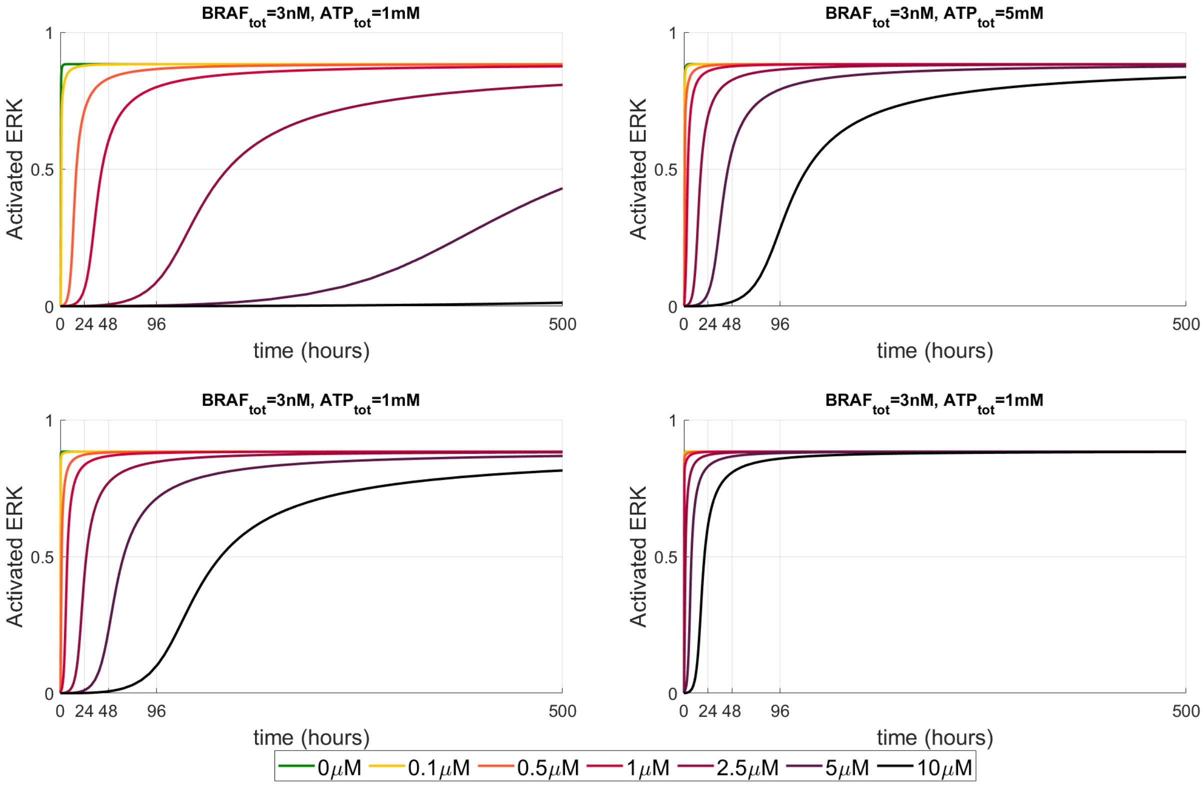

Figure SF2: Modified version of Figure 4a in the main manuscript, with a time span of 0 to 500 hours. Dabrafenib monotherapy doses are indicated by the legend at the bottom of the Figure.

References

[1] *MATLAB version 9.7.0.1471314 (R2019b) Update 7*. The Mathworks, Inc. Natick, Massachusetts, 2019.

[2] C. Y. Huang and J. E. Ferrell. “Ultrasensitivity in the mitogen-activated protein kinase cascade”. In: *Proc Natl Acad Sci USA* 93.19 (1996), pp. 10078–10083.

[3] W. S. VanScyoc, G. A. Holdgate, J. E. Sullivan, and W. H. Ward. “Enzyme kinetics and binding studies on inhibitors of MEK protein kinase”. In: *Biochemistry* 47.17 (2008), pp. 5017–5027.

[4] G. Hatzivassiliou et al. “RAF inhibitors prime wild-type RAF to activate the MAPK pathway and enhance growth”. In: *Nature* 464.7287 (2010), pp. 431–435.

[5] FDA (US Food and Drug Administration). “Reference ID: 4255750”. In: *Available on: https://www.accessdata.fda.gov/drugsatfda_docs/label/2018/202806s008lbl.pdf* (2018).

[6] T. R. Rheault et al. “Discovery of Dabrafenib: A Selective Inhibitor of Raf Kinases with Antitumor Activity against B-Raf-Driven Tumors”. In: *ACS Med Chem Lett* 4.3 (2013), pp. 358–362.

[7] J. Tsai et al. “Discovery of a selective inhibitor of oncogenic B-Raf kinase with potent antimelanoma activity”. In: *Proc Natl Acad Sci U S A* 105.8 (2008), pp. 3041–3046.

[8] P. Koelblinger, O. Thuerigen, and R. Dummer. “Development of encorafenib for BRAF-mutated advanced melanoma”. In: *Curr Opin Oncol* 30.2 (2018), pp. 125–133.

[9] S. J. Mansour, J. M. Candia, J. E. Matsuura, M. C. Manning, and N. G. Ahn. “Interdependent domains controlling the enzymatic activity of mitogen-activated protein kinase kinase 1”. In: *Biochemistry* 35.48 (1996), pp. 15529–15536.

[10] A. G. Gilmartin et al. “GSK1120212 (JTP-74057) is an inhibitor of MEK activity and activation with favorable pharmacokinetic properties for sustained in vivo pathway inhibition”. In: *Clin Cancer Res* 17.5 (2011), pp. 989–1000.
